# A test-retest resting, and cognitive state EEG dataset during multiple subject-driven states

**DOI:** 10.1038/s41597-022-01607-9

**Published:** 2022-09-13

**Authors:** Yulin Wang, Wei Duan, Debo Dong, Lihong Ding, Xu Lei

**Affiliations:** 1 Sleep and NeuroImaging Center, Faculty of Psychology, Southwest University, Chongqing, 400715 China; 2 Key Laboratory of Cognition and Personality (Southwest University), Ministry of Education, Chongqing, 400715 China; 3 Institute of Neuroscience and Medicine, Brain & Behaviour (INM-7), Research Centre Jülich, Jülich, Germany; 4 National Demonstration Center for Experimental Psychology Education (Southwest University), Chongqing, 400715 China

**Keywords:** Neuroscience, Research data

## Abstract

Here we present a test-retest dataset of electroencephalogram (EEG) acquired at two resting (eyes open and eyes closed) and three subject-driven cognitive states (memory, music, subtraction) with both short-term (within 90 mins) and long-term (one-month apart) designs. 60 participants were recorded during three EEG sessions. Each session includes EEG and behavioral data along with rich samples of behavioral assessments testing demographic, sleep, emotion, mental health and the content of self-generated thoughts (mind wandering). This data enables the investigation of both intra- and inter-session variability not only limited to electrophysiological changes, but also including alterations in resting and cognitive states, at high temporal resolution. Also, this dataset is expected to add contributions to the reliability and validity of EEG measurements with open resource.

## Abstract (summary)

**Table:** 

Measurement(s)	resting and cogntive state EEG
Technology Type(s)	electroencephalography (EEG)
Factor Type(s)	session
Sample Characteristic - Organism	human being

## Background & Summary

Electroencephalogram (EEG) is defined as the electrical activity, which is normally recorded at the scalp of the human brain, generated by the synchronous activity of neurons within the brain^1,2^. Given the various advantages of EEG including non-invasive, high temporal resolution, easy-to-operate, and cheap^3,4^ as a neuroimaging technique, it is surprising that there exit relatively fewer high-quality, open-access, big EEG datasets when compared to magnetic resonance imaging (MRI) datasets to enable the investigation of the brain function. Such open EEG data can help to accelerate the investigation of important scientific questions related to the functional significance and mechanisms of the EEG generation. On the other hand, these advantages of EEG make it particularly suitable for the acquisition of “big brain data”, which usually collected from a large number of participants with many times^5^.

One important concern in the development of an EEG-based method (e.g., connectivity analysis) to assess human brain function is to demonstrate that such measures have high test-retest reliability^6–9^. A number of studies have shown that the EEG is relatively stable when measured during resting states (rsEEG)^7^ or during the performance of cognitive tasks, such as oddball stimulus detection^10,11^, working memory (WM)^10–12^ and a psychomotor vigilance task (PVT)^7^. However, it remained unknown whether the EEG measured during the more continuous and purely subject-driven cognitive states^13^ will be more stable than the rsEEG. Moreover, based on the work of Shirer and colleagues^13^ to decode subject-driven cognitive states with whole-brain connectivity patterns, it is of great importance to train a classifier to differentiate the resting and cognitive states using EEG-based method. Candidate features can be power spectrum^14^, functional connectivity^15^, microstates^8^ and network measures of EEG^3^. To this end, some methodological questions may arise to establish the reliability of the trained classifier, such as “in terms of feature selections, are source-level features better than sensor-level features to differentiate the resting and cognitive states?” The following dataset allows researchers to address these questions by including both resting (eyes-open and eyes-closed) and subject-driven cognitive states (memory, music, subtraction) EEG with both short-term and long-term designs.

Another important concern in the development of an EEG-based method is to take into account the dynamic aspect of spontaneous brain fluctuations^16,17^. For instance, individual differences in mental and affective states, level of sleepiness, and content of spontaneously generated thoughts during the EEG session may contribute to the increased between-session variance that is independent of electrophysiological factors. To address these influences, this study collected a broad range of behavioural data to assess the participants’ sleep, emotion, mental health, and mind-wandering, and repeatedly measured variables of interest (e.g., the content of self-generated thoughts measured with the mini–New York Cognition Questionnaire^17^ and the Amsterdam Resting-State Questionnaire^18^). Hence, this data also allows researchers to investigate the reliability of the resting state questionnaires, for instance, which one is more reliable during the one-month interval, the mini–New York Cognition Questionnaire or the Amsterdam Resting-State Questionnaire?

We present here an EEG dataset with high time resolution acquired at both resting (eyes open and eyes closed) and subject-driven cognitive states (memory, music, subtraction) - collected from 60 participants at three time points. Several features of this dataset make it unique. First, similar to the SLIM Data Repository^19^ also acquired at Southwest University, all participants in this project are undergraduate students so that the age span is small. This reduces the individual differences of the resting and cognitive EEG that may arise with the age. Second, this dataset is optimal for addressing EEG-based methodological questions requiring reliability assessment. For example, a recent work from our lab examined the reproducibility of power spectrum, functional connectivity and network construction in both eyes-open and eyes-closed resting state EEG^3^ using part of the dataset. Based on the findings in that study, another study in our lab further compared the reliability of power spectrum, functional connectivity and network construction in resting to cognitive state EEG^20^. Interestingly, we found the mental subtraction state has the highest reproducibility among all the three task states. Third, this dataset enables investigation to decode the five EEG states (eyes-open, eyes-closed, memory, music, subtraction) using EEG-based measures (e.g., power spectrum, functional connectivity and microstates) as features. In addition to the crucial need for methods validation specific to EEG data, this dataset can also provide inspiring insights into the relation of mental state (using measures of sleep, emotion, mental health, mind-wandering, and the content of self-generated thought) to electrophysiology. For example, participants always took mini–New York Cognition Questionnaire after each of the five EEG states, is there going to be some difference regarding the content of self-generated thought between resting and cognitive states? And how is this difference corresponding to the EEG-based features?

In the follow-up sessions, we briefly describe the participant recruitment, data collection, data record, technical validation, and sharing and access policy.

## Methods

### Overall design

**Fig. 1:**
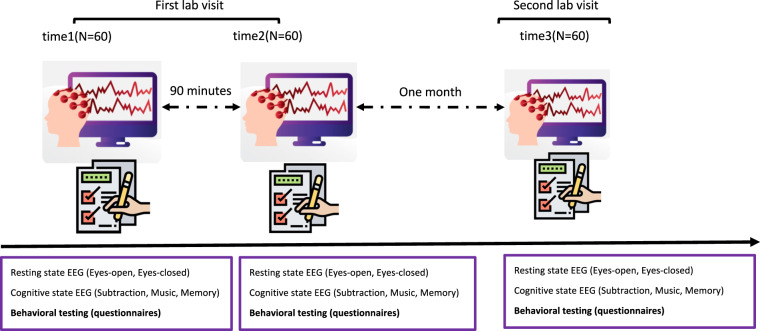
Overall study design. The dataset includes EEG data and behavioural variables measured with questionnaires at three time points.

The data collection was initiated in September 2019 and was terminated in April 2021. The general information is shown in Fig. 1. The dataset includes electroencephalogram (EEG) data from 60 participants with all three recording sessions, including the present (session 1), 90 min later (session 2), and one month later (session 3). The average age of all the participants is 20.01 years old (range 18–28) and the median is 20 years old. There are 32 females and 28 males. Part of the dataset was utilized to investigate the reproducibility of power spectrum, functional connectivity and network construction in eyes-open and eyes-closed resting-state EEG, and was published in *Journal of Neuroscience Methods*^3^.

### Participants

Participants were initially recruited through online advertisement. The inclusion criteria included: (1) right-handed; (2) Body Mass index (BMI) lower than 28; (3) go to sleep no later than 00:30 am. The exclusion criteria included: (1) current psychiatric disorders and neurological disorders; (2) use of psychiatric drugs within the three months prior to the recording; or (3) a history of head trauma. No alcoholic, caffeinated food or drink was allowed on the EEG recording date. Every participant was compensated for participation (around 30 dollars). Written informed consent was obtained from all the participants after a detailed explanation of the study protocol. The conduction of all the experiments were in accordance with the Declaration of Helsinki. This study was approved by the Review Board of the Institute of Southwest University.

### Experimental design

As indicated in Fig. 1, for each subject, a series of behavioral data was collected. These data include general demographics for the EEG study (e.g., gender and age), widely used questionnaires that investigate participants’ sleep, mind wandering, mental health and emotion. We released part of the behavioral data with the EEG data. Other variables will be considered to be made public in the future or when requested by researchers via e-mail. The detailed list of the behavioral data is provided in the testing procedure below. The information regarding the released behavioral variables is summarized in Table 1.

**Table 1:** Released behavioural data.

Name	Type	Key variables	Reference
Self-rating Anxiety Scale (SAS)	Repeated 2 times	Total score	Zung, 1971
Self-rating Depression Scale (SDS)	Repeated 2 times	Total score	Zung, 1965
Epworth Sleeping Scale (ESS)	Repeated 2 times	Total score	Johns^23^
Amsterdam Resting-State questionnaire 2.0 (ARSQ)	Repeated 3 times	Factor scores	Diaz *et al*.^14^
Karolinska Sleepiness Scale (KSS)	Repeated 3 times	Total score	Akerstedt and Gillberg, 1990
Positive and Negative Affect Schedule (PANAS)	Repeated 3 times	Positive and negative affect scores	Watson *et al*. 1998
Mini New York Cognition Questionnaire (mini-NYC-Q)	Repeated 15 times	Factor scores	Gorgolewski *et al*. 2015

### Testing procedure

Each participant was invited to the lab twice exactly one month apart (therefore visits were matched in terms of time of the day and day of the week). Participants were instructed to sit quietly in front of the computer in a comfortable position. Participants received the testing protocol twice with 90 minutes apart during their first visit and had the same testing protocol for a third time during their second visit. The data acquired is summarized in Fig. 1, and the order of tasks/measurements was the following (details of each released test are described in the next section):

### First lab visit (time 1)

1. At the behavioural testing room
  Participants first received the briefing about the experiment and signed the informed consent. They then filled in some demographic questionnaires measuring ethnics, gender, age, eyesight, family medical history et cn. Next, participants also filled in the Self-rating Anxiety Scale (SAS), Self-rating Depression Scale (SDS) and Epworth Sleeping Scale (ESS).
2. At the EEG recording room

Participants first had a five minute of eyes-open rest EEG recording and filled in the Mini New York Cognition Questionnaire (mini NYC-Q). Then they continued to have a five minute of eyes-closed rest EEG and filled in the mini NYC-Q. Immediately after the resting-state EEG recording sessions, they filled in the Amsterdam Resting-State questionnaire 2.0 (ARSQ), Stanford Sleepiness Scale (SSS), Karolinska Sleepiness Scale (KSS), Positive and Negative Affect Schedule (PANAS). As a follow-up, participants then received the three cognitive state EEG recordings. ﻿To make sure the participants were taking part in the experiment carefully, they also received relative questionnaires after each cognitive state. Moreover, they would have got probed during the specific cognitive state task (e.g., pause and let them say the current number in the Subtraction task). Similar to the resting state EEG recording sessions, participants also filled in the mini NYC-Q right after each cognitive state EEG recording session.

### First lab visit (time 2)

1. At the EEG recording room- same as time 1

After 90 minutes as indicated in Fig. 1, participants had the same testing procedure as time 1 in the EEG recording room.

### Second lab visit

1. At the behavioural testing room- ﻿differences from the first visit in bold:
  As indicated in Fig. 1, participants came to the lab one month later. They again filled in the Self-rating Anxiety Scale, Self-rating Depression Scale and Epworth Sleeping Scale. Different from their first lab visit, they also filled in **Pittsburgh Sleep Quality Index (PSQI), Munich Chrono Type Questionnaire 2.0 (MCTQ), Reduced version of Morningness-Eveningness Questionnaire (rMEQ), Eysenck Personality Questionnaire (EPQ) and Sleep Hygiene of College Students**.
2. At the EEG recording room- same as time 1

As indicated in Fig. 1, participants had the same testing procedure as their first lab visit in the EEG recording room.

### Released behavioural tests

#### Self-rating Anxiety Scale (SAS)

The SAS is a 20-item, self-administered survey to measure anxiety levels, based on scoring in four groups of manifestations: cognitive, autonomic, motor and central nervous system symptoms^21^. Participants indicate how much each statement applies to him or her within a period of one or two weeks prior to taking the test. Each question is scored on a Likert-type scale of 1–4 (“a little of the time”, “some of the time”, “good part of the time”, “most of the time”). Overall assessment is done by total score. SAS was presented to participants upon both their first and second lab visit. The aim is to check whether the anxious status of a participant will change for one month interval.

#### Self-rating Depression Scale (SDS)

The SDS is a 20-item, self-administered survey to quantify the depressed status of a patient/participant, based on scoring in four groups of four common characteristics of depression: the pervasive effect, the physiological equivalents, other disturbances, and psychomotor activities^22^. Each question is scored on a Likert-type scale of 1–4 (“a little of the time”, “some of the time”, “good part of the time”, “most of the time”). Overall assessment is done by total score. SDS was also presented to participants upon both their first and second lab visit. The aim is to check whether the depressive status of a participant will change for one month interval.

#### Epworth Sleeping Scale (ESS)

The ESS is a self-administered questionnaire with eight questions measuring daytime sleepiness^23^. Participants are asked to rate, on a 4-point scale (0–3), their usual chances of falling asleep while engaged in eight different activities. The scores for the eight questions are added together to obtain a single number. ESS was also presented to participants upon both their first and second lab visit. The aim is to check whether the daytime sleepiness status of a participant will change for one month interval.

#### Amsterdam Resting-State questionnaire (ARSQ) 2.0

ARSQ 2.0 quantifies mind wandering along ten dimensions: “Discontinuity of Mind”, “Theory of Mind”, “Self”, “Planning”, “Sleepiness”, “Comfort”, and “Somatic Awareness”, “Health Concern”, “Visual Thought”, and “Verbal Thought^18^”. All 54 statements were scored on a five-point Likert-type scale (1–5) with the labels “Completely Disagree”, “Disagree”, “Neither Agree nor Disagree”, “Agree”, and “Completely Agree”. ARSQ 2.0 was presented to participants every time after they had the resting state EEG recordings. Hence, each participant took ARSQ 2.0 three times during the whole experiment. Notably, the item order for the ARSQ 2.0 was randomized, except for the last two validation items (“I had my eyes closed” and “I was able to rate the statements). The aim is to validate the test-retest reliability of the ARSQ 2.0 for both short-term and long-term intervals.

#### Karolinska Sleepiness Scale (KSS)

KSS measures the subjective level of sleepiness at a particular time during the day^24^. On this 9-point scale (1 = extremely alert, 3 = alert, 5 = neither alert nor sleepy, 7 = sleepy – but no difficulty remaining awake, and 9 = extremely sleepy – fighting sleep) participants indicate which level best reflects the psycho-physical sate experienced in the last 10 min. The KSS was presented to the participants at all the three time points (Fig. 1) when they were at the EEG recording room.

#### Positive and Negative Affect Scale (PANAS)

PANAS is a self-report questionnaire that consists of two 10-item scales to measure both positive and negative affect^25^. Each item is rated on a 5-point scale of 1 (not at all) to 5 (very much). The PANAS was also presented to the participants at all the three time points (Fig. 1) when they were at the EEG recording room.

#### Mini New York Cognition Questionnaire (mini NYC-Q)

The mini version of the New York Cognition Questionnaire is an adaptation of the full version of New York Cognition Questionnaire which consists of 12 items^17^. Participants had to rate each statement/item on a 11-point scale ranging from ‘Completely did not describe my experience’ (score 0) to ‘Completely described my experience’ (score 10). The questionnaire was presented immediately after each resting sate EEG, and each cognitive state EEG recording at all three time points. Finally, each participant was measured 15 times of mini NYC-Q during the whole experiment.

### Released EEG recordings

#### EEG resting state

During resting-state EEG recording, participants were instructed to view a fixation point for five minutes (Eyes Open) and then close eyes for another five minutes (Eyes Closed). They needed to keep still, quiet, and relaxed as much as they can, and try to avoid blinking^3^ for Eyes Open (EO) session and stay awake for Eyes Closed (EC) session.

#### EEG cognitive state

The present experiment consisted of three subject-driven cognitive states: retrieval of recent episodic memories, serial subtractions, and (silent) singing of music lyrics^13^. The order of the three cognitive tasks was counterbalanced during the recording. For the **memory** task, participants were asked to recall the events of the day from when they awoke until they arrived at the lab. For the **music** task, participants were asked to sing their favourite songs in their head. For the **subtraction** task, participants were asked to count backwards from 5000 by 7s. Participants were instructed to keep their eyes closed during each of the self-driven cognitive states. They could repeat the task (e.g., they should sing the same song repeatedly in their head) for multiple times if they finished with less than five minutes. In addition, participants received corresponding questionnaires after each cognitive task state to check whether they stayed in the task state as instructed.

#### EEG acquisition

Continuous scalp EEG was recorded by either a 63 or a 64 Ag/AgCl active electrodes mounted within an elastic cap, based on the extended 10–20 international electrode placement system (Brain Products GmbH, Steing- rabenstr, Germany) for each experimental session. Two of these channels were used to record electrooculograms and the FCz was utilized as the online reference channel. The sampling rate was 500 Hz and the electrode impedance was kept below 5 kΩ after careful preparation.

#### Segmentation of EEG data

To unify the channels from the different size of caps during EEG recording, we first reconstructed the channels as 62 for all the 180 data files (three sessions × 60 participants). The recorded EEG data of each experimental session was further segmented into five parts according to the markers of the onset time of the state, which is corresponding to the five states (eyes-closed, eyes-open, memory, music, and subtraction). The order of the five states during data recording was given with the “participants.tsv” file. Of note, for the EEG data during the eyes-open state, independent component analysis (ICA) was performed, and then ICLabel was used to identify the eye movement component for removal and finally the EEG data was reconstructed for sharing with OpenNeuro.

## Data Records

**Fig. 2:**
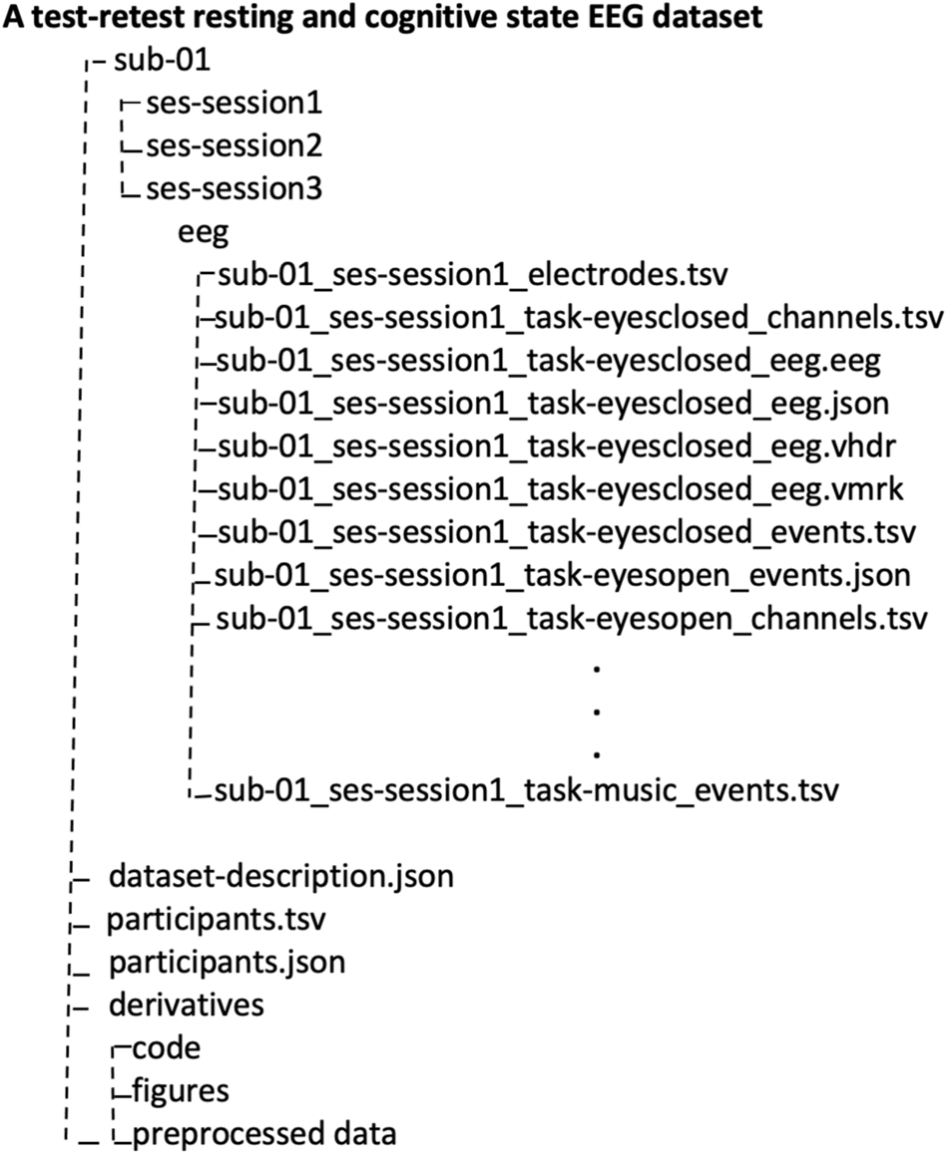
The structure of the dataset in BIDS format.

All the segmented data are available in BIDS format^26,27^, and uploaded separately to OpenNeuro site (https://openneuro.org) under the name: ***A test-retest resting and cognitive state EEG dataset***^28^. The main folder of this Dataset (available at: https://openneuro.org/datasets/ds004148/versions/1.0.1) contains 60 folders, one for each participant, and one derivatives folder containing the pre-processed data and code to reproduce the figures and four files: (i) “data-description.json” that describes the dataset and contains information about where and when the data are registered and (ii) “participants.tsv” that contains information about the participants such as sex and age, as well as the behavioural data measured with questionnaires mentioned above and (iii) “participants.json” that describes all the columns presented in “participants.tsv” file and iiii) “README” that describes the general information about the dataset, including the contact information. Each participant’s folder contains three subfolders for the three time points, the three subfolders contain the EEG data, electrodes, channels, events, etc … for the five resting and cognitive states (See Fig. 2).

## Technical Validation

**Fig. 3:**
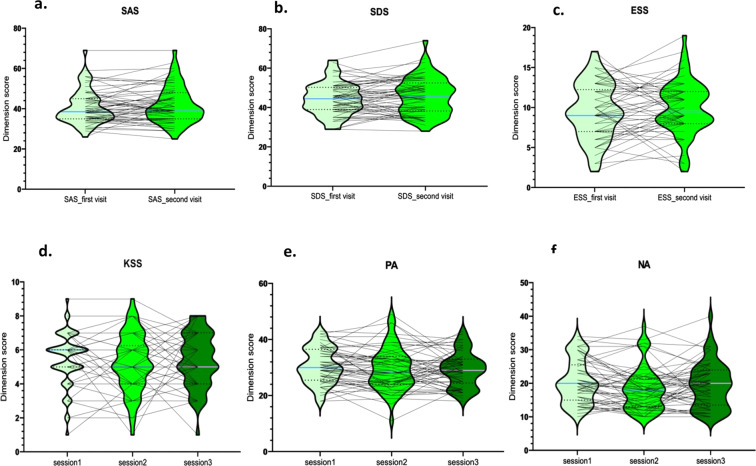
Distribution of SAS, SDS, as well as ESS across participants and between visits. Each line corresponds to one participant. SAS = Self-rating Anxiety Scale; SDS = Self-rating Depression Scale; ESS = Epworth Sleeping Scale; KSS = Karolinska Sleepiness Scale; PA = Positive Affect; NA = Negative Affect.

To investigate whether the anxious status of a participant will change for one month interval, we have plotted the overall score of the SAS during both their first and second lab visit (see Fig. 3a). It turned out that the anxious status of a participant did not change for one month interval, *t* (57) = −1.38, *p* = 0.17.

To investigate whether the depressive status of a participant will change for one month interval, we have plotted the overall score of the SDS during both their first and second lab visit (see Fig. 3b). It turned out that the depressive status of a participant did not change for one month interval, *t* (57) = −1.56, *p* = 0.12.

To investigate whether the daytime sleepiness status of a participant will change for one month interval, we have plotted the overall score of the ESS during both their first and second lab visit (see Fig. 3c). It turned out that the daytime sleepiness of a participant did not change for one month interval, *t* (57) = −1.11, *p* = 0.27.

**Fig. 4:**
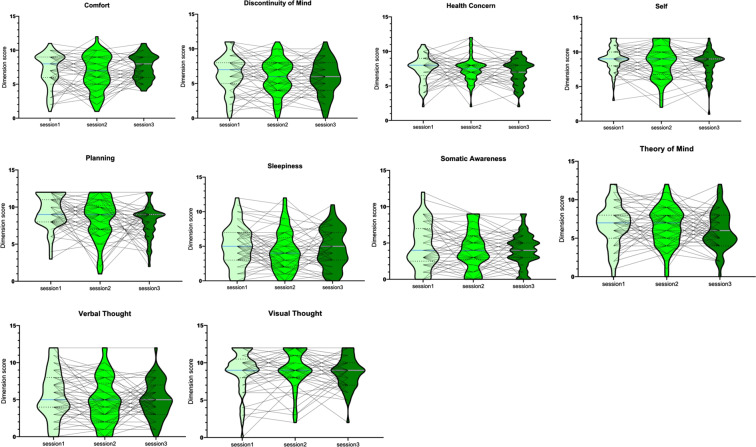
Evolution of the content of ARSQ 2.0 over the course of the experiment. Each line corresponds to one participant.

To assess the variance of the self-reported content of mind wandering along ten dimensions: “Discontinuity of Mind”, “Theory of Mind”, “Self”, “Planning”, “Sleepiness”, “Comfort”, and “Somatic Awareness”, “Health Concern”, “Visual Thought”, and “Verbal Thought”, we have plotted the evolution of the answers for ARSQ 2.0 over the three time points (see Fig. 4). There is a high variance between as well as within participants, consistent with the nature of mind wandering. It appeared that participants experienced significant less “planning” at session3 compared to session1(*F* (2,167) = 4.91, *p* = 0.008) and participants experienced significant more “Verbal Thought” at session3 compared to session1(*F*(2,167) = 28.56, *p* < 0.001). The other eight dimensions did not change across the three experimental sessions (all *p*s > 0.05).

To assess the variance of Karolinska Sleepiness Scale, we have plotted the evolution of the answers for KSS over the three time points (see Fig. 3d). It turned out that the sleepiness status of a participant did not change across the three experimental sessions, *F*(2, 169) = 0.25, *p* = 0.78.

To assess the variance of Positive and Negative Affect Scale, we have plotted the evolution of the answers for Positive Affect (see Fig. 3e) and Negative Affect (see Fig. 3f) over the three time points. It turned out that both the Positive Affect (*F* (2, 169) = 0.72, *p* = 0.49) and Negative Affect (*F*(2, 169) = 1.57, *p* = 0.21) of a participant did not change across the three experimental sessions.

### mini NYC-Q

**Fig. 5:**
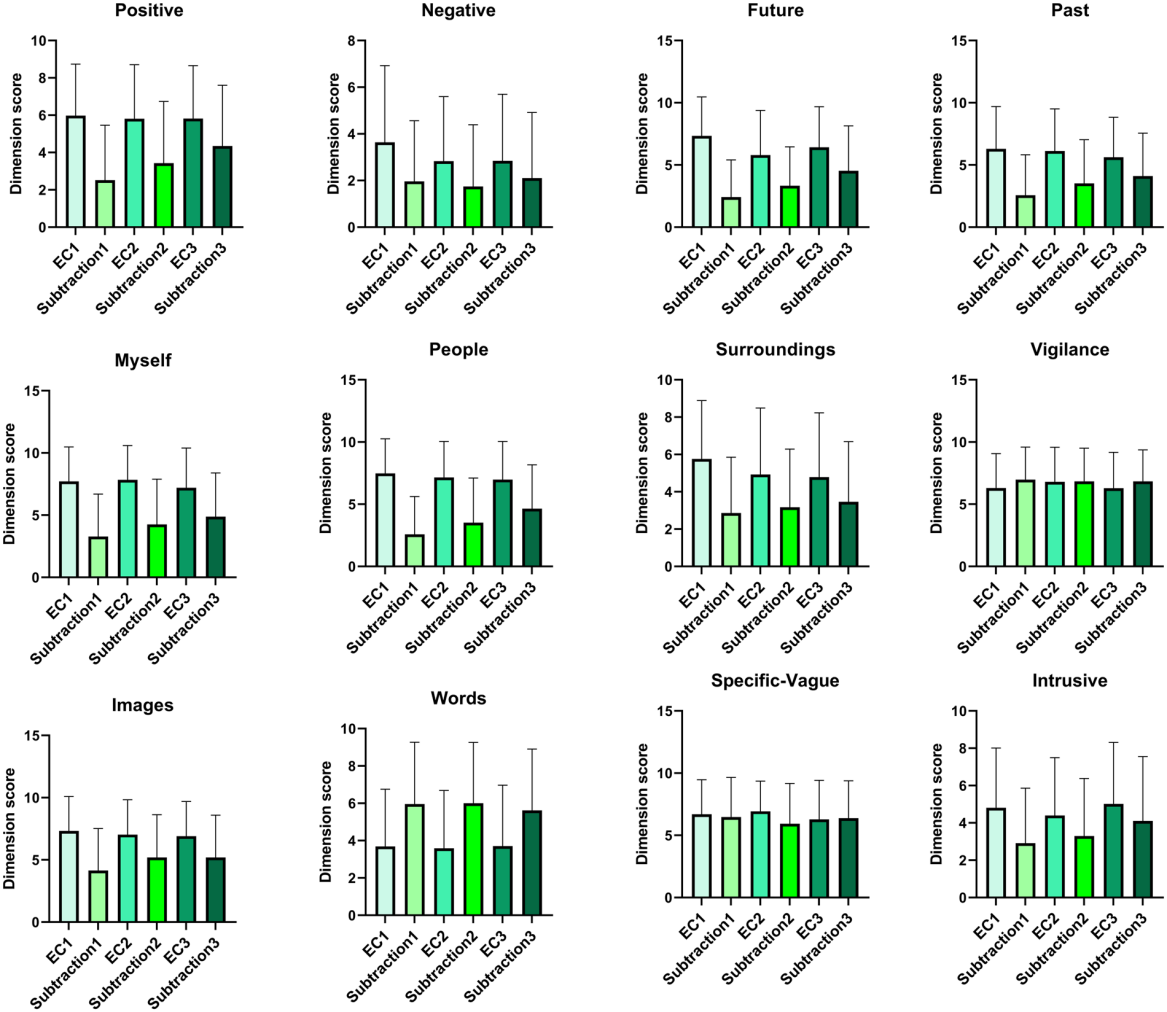
Evolution of the content of self-generated thoughts and vigilance over the course of the experiment under the eyes-closed and subtraction states. Each line corresponds to one participant. First two time points correspond to the first session—the middle two to the second session—the last two to the third session. EC: eyes-closed.

To assess the variance of the self-reported content of self-generated thoughts measured by the Mini New York Cognition Questionnaire, we have plotted the evolution of the answers for the eyes-closed and subtraction states over the period of the two visits (see Fig. 5). There is a high variance between as well as within participants, consistent with the nature of mind wandering. We have noticed that the answers for all the items did not differ among the experimental sessions regardless of the states (see Table 2 for the statistics). The answers for items *positive*, *negative*, *future*, *past*, *myself*, *people*, *surroundings*, *images* and *intrusive* was higher under the eyes-closed compared to the subtraction state while the opposite pattern was found for *words* (see Table 2 for the statistics). The answers for item *vigilance* and *specific-vague* did not differ between the eyes-closed and subtraction states (see Table 2 for the statistics). Participants’ thoughts were getting more and more about something positive, involved more future events and other people under the subtraction state while remained unchanged under the eyes-closed state through the three time points (see Table 2 for the statistics).

**Table 2:** The repeated measurements of the answers for the mini NYC-Q items under the eyes-closed and subtraction states over the three time points.

Items	Session Main Effect	State Main Effect	Session × State interaction Effect
Positive (Item1)	*F* (2, 55) = 0.15, *p* = 0.15	*F* (1, 56) = 59.67, *p* < 0.001	*F* (2, 55) = 7.56, *p* = 0.001
Negative (Item2)	*F* (2, 55) = 1.48, *p* = 0.24	*F* (1, 56) = 13.75, *p* < 0.001	*F* (2, 55) = 1.33, *p* = 0.27
Future (Item3)	*F* (2, 55) = 2.08, *p* = 0.14	*F* (1, 56) = 72.00, *p* < 0.001	*F* (2, 55) = 8.04, *p* < 0.001
Past (Item4)	*F* (2, 55) = 0.70, *p* = 0.50	*F* (1, 56) = 51.52, *p* < 0.001	*F* (2, 55) = 3.75, *p* = 0.03*
Myself (Item5)	*F* (2, 55) = 2.00, *p* = 0.15	*F* (1, 56) = 83.73, *p* < 0.001	*F* (2, 55) = 4.03, *p* = 0.02*
People (Item6)	*F* (2, 55) = 2.62, *p* = 0.08	*F* (1, 56) = 96.51, *p* < 0.001	*F* (2, 55) = 7.74, *p* = 0.001
Surroundings (Item7)	*F* (2, 55) = 0.23, *p* = 0.79	*F* (1, 56) = 37.97, *p* < 0.001	*F* (2, 55) = 2.83, *p* = 0.07
Vigilance (Item8)	*F* (2, 55) = 0.23, *p* = 0.47	*F* (1, 56) = 3.14, *p* = 0.08	*F* (2, 55) = 21.45, *p* = 0.24
Images (Item9)	*F* (2, 55) = 1.59, *p* = 0.21	*F* (1, 56) = 37.97, *p* < 0.001	*F* (2, 55) = 3.41, *p* = 0.04*
Words (Item10)	*F* (2, 55) = 0.10, *p* = 0.91	*F* (1, 56) = 26.92, *p* < 0.001	*F* (2, 55) = 0.37, *p* = 0.69
Specific-Vague (Item11)	*F* (2, 55) = 0.38, *p* = 0.68	*F* (1, 56) = 1.912, *p* = 0.17	*F* (2, 55) = 1.40, *p* = 0.26
Intrusion (Item12)	*F* (2, 55) = 1.69, *p* = 0.19	*F* (1, 56) = 27.40, *p* < 0.001	*F* (2, 55) = 1.61, *p* = 0.21

Note. *Donates p values don’t survive the multiple comparisons with false discovery rate.

### Pre-processing of the EEG data

The same pre-processing procedure in our previous study was applied^20^. The pre-processing of the original EEG signal contained five steps, which was implemented in EEGLAB (version 2019_1, http://sccn.ucsd.edu). In the first step (Re-reference), the raw data were re-referenced to a common average reference and filtered using a symmetric Finite Impulse Response filter with frequency band of 0.3–45 Hz. In the second step, EEG signals were visually inspected (If a channel contains 1/3 or higher ratio of the problematic trials, it will be considered as a bad one), and it turned out no bad channels were removed. And then we used linear interpolation to reconstruct missing data (replaced by the average of the surrounding nearest neighbour channels). Then the third step involved the re-reference of the data to a common average reference. After that, the EEG signal was segmented into 4-s epochs and then all bad epochs were manually screened for removal. It turned out no bad epochs were identified in this dataset. The fourth step is running independent component analysis (ICA) within EEGLAB for the eyes-open state, some independent components were marked as artifacts (e.g., eye blinks, eye movements). On average, 0.978 (±0.146) components of each EEG session during eyes-open were identified as artifacts.

### Power spectrum of five states at three sessions

**Fig. 6:**
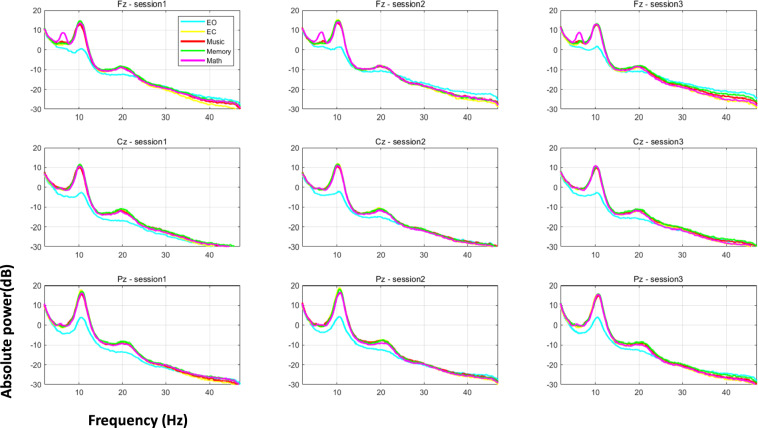
Averaged Power spectrum across all the participants of five states (eyes-open, eyes-closed, music, memory, subtraction) and three experimental sessions at Fz, Cz and Pz electrodes. EO: eyes-open; EC: eyes-closed; Math: subtraction.

For the preprocessed dataset, we then calculated the power spectrum of each electrode using Welch’s method (window and segment length: 4 s, non-overlap). The absolute power of each electrode was log- transformed to calculate the power spectrum (1 dB = 10 × log μV^2^). Then the spectrum was averaged within each frequency band to obtain the power value of seven rhythms: delta (1–4 Hz), theta (4–8 Hz), alpha1 (8–11 Hz), alpha2 (11–13 Hz), beta1 (13–20 Hz), beta2 (20–30 Hz) and gamma (30–45 Hz). A Power spectrum was averaged over Fz, Cz and Pz electrodes for illustration, and the mean and standard deviation was calculated for the participants in each state. Notably, the maximum power of the EEG signal spectrum of the five states was concentrated in the low frequency (around 0.3–13 Hz), and there was a peak around 10 Hz (see Fig. 6). Moreover, the spectrum of the four states of eyes-closed, subtraction, music, and memory are particularly similar, all showing a power peak above 10 dB at around 10 Hz while the eyes-open state seemed to encounter a lower power.

To test whether there is a difference of alpha power (8–13 Hz) between the eyes-open and the other four states, and how this difference will evolve with the experimental sessions, we have extracted the alpha power spectrum of the three electrodes (Fz, Cz and Pz) for all the participants at the five states of the three experimental sessions. A 3 × 5 (Experimental session [session 1, session2, session3] × state [eyes-open, eyes-closed, music, memory, subtraction]) ANOVA was conducted for the alpha power of Fz, Cz and Pz separately. For Fz (*F*(4.56) = 94.92, *p* < 0.001), Cz (*F*(4.56) = 110.08.92, *p* < 0.001) and Pz (*F*(4.56) = 71.55, *p* < 0.001), there was a big difference between eyes-open and the other four states. The alpha power of eyes-open was significantly lower than the other states. The above phenomena were stable across the three sessions, implied good reproducibility in all five states.

### Power distribution of seven rhythms in five states

**Fig. 7:**
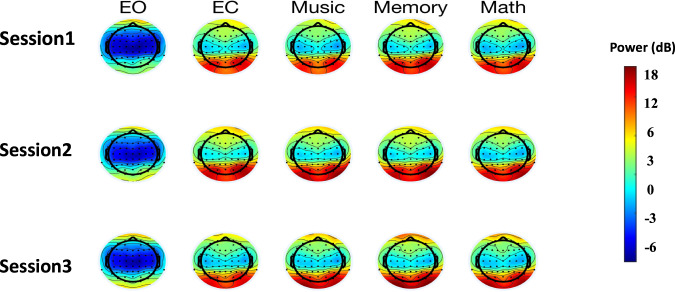
The power distribution of alpha rhythm in five resting and cognitive states. EO: eyes-open; EC: eyes-closed; Math: subtraction.

The topographies of alpha (8–13 Hz) were illustrated in Fig. 7. The high-power area of the alpha rhythm was mainly around the occipital lobe for the states of eyes-closed, memory, music, and subtraction. In comparison, the alpha rhythm was mainly manifested as low energy in the parietal lobe for the eyes-open state.

## Usage Notes

Some of the most common software packages that can be used for analysing these data are freely available, and include FieldTrip^29^ (http://fieldtrip.fcdonders.nl/), MNE^30^ (http://martinos.org/mne/), Brainstorm^31^ (http://neuroimage.usc.edu/brain- storm), EEGLAB^32^ (http://sccn.ucsd.edu/eeglab/), and EEGNET^33^ (https://sites.google.com/site/eegnetworks/), Automagic^34^ (https://github.com/methlabUZH/automagic). EEGLAB was used by our lab to pre-process this dataset and made the figures.

This dataset has multiple potential uses for cognitive neuroscience and for methodological development in EEG analysis, such as:

1. compare the reliability of power spectrum, functional connectivity and network measures in resting and subjective-driven cognitive state EEG.
2. decode the five EEG states (eyes-open, eyes-closed, memory, music, subtraction) using EEG-based features (e.g., power spectrum, functional connectivity and microstates).
3. Investigate the relation of mental state (using measures of sleep, emotion, mental health, mind-wandering, and the content of self-generated thought) to electrophysiology.

## Data Availability

The code used to convert the test-retest resting, and cognitive state EEG dataset for sharing with OpenNeuro was referred to the conversion of the EEG sedation dataset for sharing in BIDS, which is public available at https://www.fieldtriptoolbox.org/workshop/madrid2019/bids_sedation/. The BIDS background is explained on http://bids.neuroimaging.io, details on the specification can be found on the paper titled “EEG-BIDS, an extension to the brain imaging data structure for electroencephalography”, which was published in Scientific Data.
